# An affordable and easy-to-use tool for automatic fish length and weight estimation in mariculture

**DOI:** 10.1038/s41598-022-19932-9

**Published:** 2022-09-19

**Authors:** Nicolò Tonachella, Arianna Martini, Marco Martinoli, Domitilla Pulcini, Andrea Romano, Fabrizio Capoccioni

**Affiliations:** Consiglio Per La Ricerca in Agricoltura E L’Analisi Dell’Economia Agraria (CREA), Centro “Zootecnia E Acquacoltura”, Via Salaria 31, 00015 Monterotondo, Rome, Italy

**Keywords:** Ichthyology, Marine biology, Optical imaging

## Abstract

Common aquaculture practices involve measuring fish biometrics at different growth stages, which is crucial for feeding regime management and for improving farmed fish welfare. Fish measurements are usually carried out manually on individual fish. However, this process is laborious, time-consuming, and stressful to the fish. Therefore, the development of fast, precise, low cost and indirect measurement would be of great interest to the aquaculture sector. In this study, we explore a promising way to take fish measurements in a non-invasive approach through computer vision. Images captured by a stereoscopic camera are used by Artificial Intelligence algorithms in conjunction with computer vision to automatically obtain an accurate estimation of the characteristics of fish, such as body length and weight. We describe the development of a computer vision system for automated recognition of body traits through image processing and linear models for the measurement of fish length and prediction of body weight. The measurements are obtained through a relatively low-cost prototype consisting of a smart buoy equipped with stereo cameras, tested in a commercial mariculture cage in the Mediterranean Sea. Our findings suggest that this method can successfully estimate fish biometric parameters, with a mean error of ± 1.15 cm.

## Introduction

Aquaculture provides two-thirds of the world’s aquatic products^1^. It represents a key source of high-quality proteins for humans and has become one of the fastest-growing industries in global food production^1–3^. Along with the growth of the world population, global aquaculture production is expected to increase swiftly to meet demand in the forthcoming years^3^. However, the rapid growth of aquaculture production systems has led to several concerns, such as fish overfeeding, disease outbreaks, water pollution, and related environmental issues. These matters contribute to determining the social acceptability of the sector. For these reasons, the industry and the academic community are requested to seek strategies to increase productivity and efficiency of the aquaculture systems, while controlling the negative impacts^4^ and improving the environmental, economic, and social sustainability.

In recent decades, the use of engineering, smart technologies (e.g. sensors such as probes and optical systems for water and fish monitoring), and statistical analyses have gradually gained traction in the aquaculture sector; the so-called “precision aquaculture” applies control engineering principles to fish production to improve farmers' monitoring and management of fish farms^5^. As part of the green revolution, precision tools applied in the aquaculture systems will help solve problems of global overfishing by improving productivity and sustainability^1,3^, and create a more environmentally responsible aquaculture industry. Indeed, the management of land-based and off-shore aquaculture facilities requires an accurate periodical estimation of fish length and weight to monitor fish growth, thus allowing calibration of feed administration and assessment of fish health. Length and biomass estimation is usually conducted manually on a reduced fish subsample or derived through calculations based on feed administration. Manual measurements are weather-dependent, time-consuming, and work-intensive for fish farmers, and, above all, they are invasive and stressful to fish^4,6,7^. In addition, size estimates based on measurements of a limited number of specimens may not be sufficiently consistent and have a high margin of bias. Inaccurate length and biomass estimations in fish farming can result in overfeeding, and consequently higher production costs and greater environmental impact^2,8^. In contrast to traditional methods, “precision farming” methods are not affected by weather or time constraints, are non-invasive and non-stressful for the reared animals, and can help aquaculture facilities in optimizing their capital investment, feeding protocols, and harvesting schedule^4,5^. Therefore, fast and safe alternatives to measure fish growth traits would be extremely helpful to farmers and biologists.

Computer vision deals with how computers can gain high-level understanding from digital images or videos. It can be used as an effective non-invasive technique for estimating animal biometrics, attracting the interest of researchers and farmers, as photos of the specimens can be captured and used to measure different body traits remotely^4,9–11^. The images captured by the cameras are analyzed by Artificial Intelligence (AI) algorithms to automatically obtain parameters such as body length, width or weight. Computer vision enables precise and reliable remote monitoring and automatic control of fish biological indicators, which are crucial for the correct time- and cost-efficient management of the farm. Fish length and mass estimation methods based on computer vision usually involve three steps: fish images are preprocessed; fish features are extracted from images and feature values, like body length, calculated; and the feature values are fitted to construct a prediction model to realize the estimation of fish mass^7^.

To the best of the authors’ knowledge, most of the efforts in using computer vision systems in aquaculture are associated with the categorization, shaping, behavior, count, or estimation of fish body size (mostly length)^11–14^, with only a few studies focusing on measuring body area or predicting body weight^4,15–17^; Costa et al.^18^ and Torisawa et al.^19^ estimated large bluefin tunas' lengths (ranging from 0.60 to 1.8 m) using a similar underwater twin camera module with an error of 2% and 5% of the fish's actual length, respectively.

Here we present a low-cost prototype system (“smart buoy”) tested in a commercial mariculture cage on Capraia Island (Italy). The buoy, equipped with a multi-parametric probe and stereoscopic cameras, is capable of capturing images of fish; these are then automatically analyzed by exploiting the cognitive capabilities of AI and computer vision algorithms. The technology allowed achieving automatic length estimation of cultured gilthead seabream (*Sparus aurata*, L.) at the end of the on-growing period.

## Results

### Distance-translation relationship

**Figure 1:**
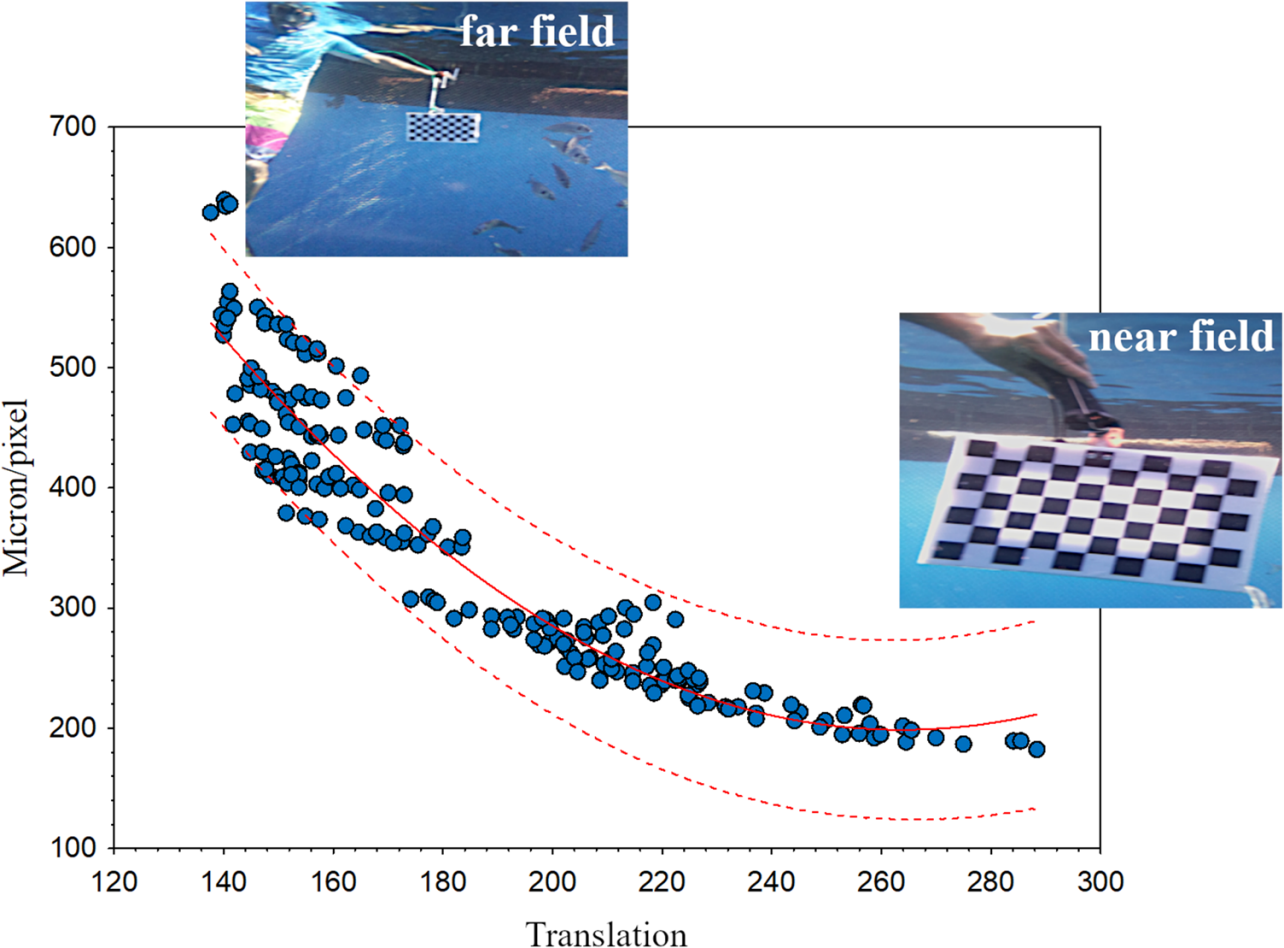
Scatterplot of the mean translation of the landmarks (n = 208) and their corresponding micron/pixel ratio (cubic polynomial fitting ± 0.95 confidence limits).

A relationship between landmarks translation and the corresponding distance from the stereo camera is shown in Fig. 1. On the y-axis the distance (in micron/pixel) between the landmarks (4 pairs for each image) selected on the chessboard is represented; the x-axis shows the average translation of each pair of landmarks on the chessboard between the two stereo images. As shown in the scatterplot, a cubic polynomial model of the two variables shows a strong correlation (R^2^ = 0.899, p = 0.00). This can therefore be used to estimate the micron/pixel ratio of an object showing a specific translation of its landmarks. The minimum pixel translation considered to be consistent with the technical characteristics of the stereo camera (e.g., the distance between the two lenses and focal length) has been set to 140 pixels, since the translation of extremely distant targets is undetectable.

### Measurement error estimation

A total of 68 measurements obtained from 17 images of fish silhouettes resulted in a mean absolute percentage error (MAPE) of ± 1.15 cm, corresponding to ± 5.5% in the fish standard length (SL) estimations.

### AI estimation of fish length and weight

The AI algorithm identified a total of 272 fish from 76 images of shoals captured within the cage. The images of 148 fish were discarded as the translation fell below the 140-pixel threshold. The remaining 124 fish were automatically processed for SL estimation (AI measurements). As a control sample, 190 seabreams were caught from the same cage where the study was carried out. Length and weight measurements were manually determined to obtain a length-to-weight relationship (W = 0.1342SL^2.5465^; R^2^ = 0.755) and to assess disparities with AI estimated measurements. This relationship was then used to obtain the fish mass from the estimated AI lengths. The mean length and weight ± SD of fish obtained from field samplings and those estimated by the AI approach are reported in Tab. 1. Mean differences, expressed as a percentage, were ± 3.0 and ± 3.6% for standard length and weight, respectively.

**Table 1:** Field and AI measurements of mean fish standard length and weight. Mean differences are calculated as the percentage between the two different approaches.

	n	Standard length (cm)	Weight (g)
Sampled	190	27.1 ± 1.6	606 ± 103
AI estimated	124	26.3 ± 4.4	585 ± 239
Mean difference (%)		3.0%	3.6%

**Figure 2:**
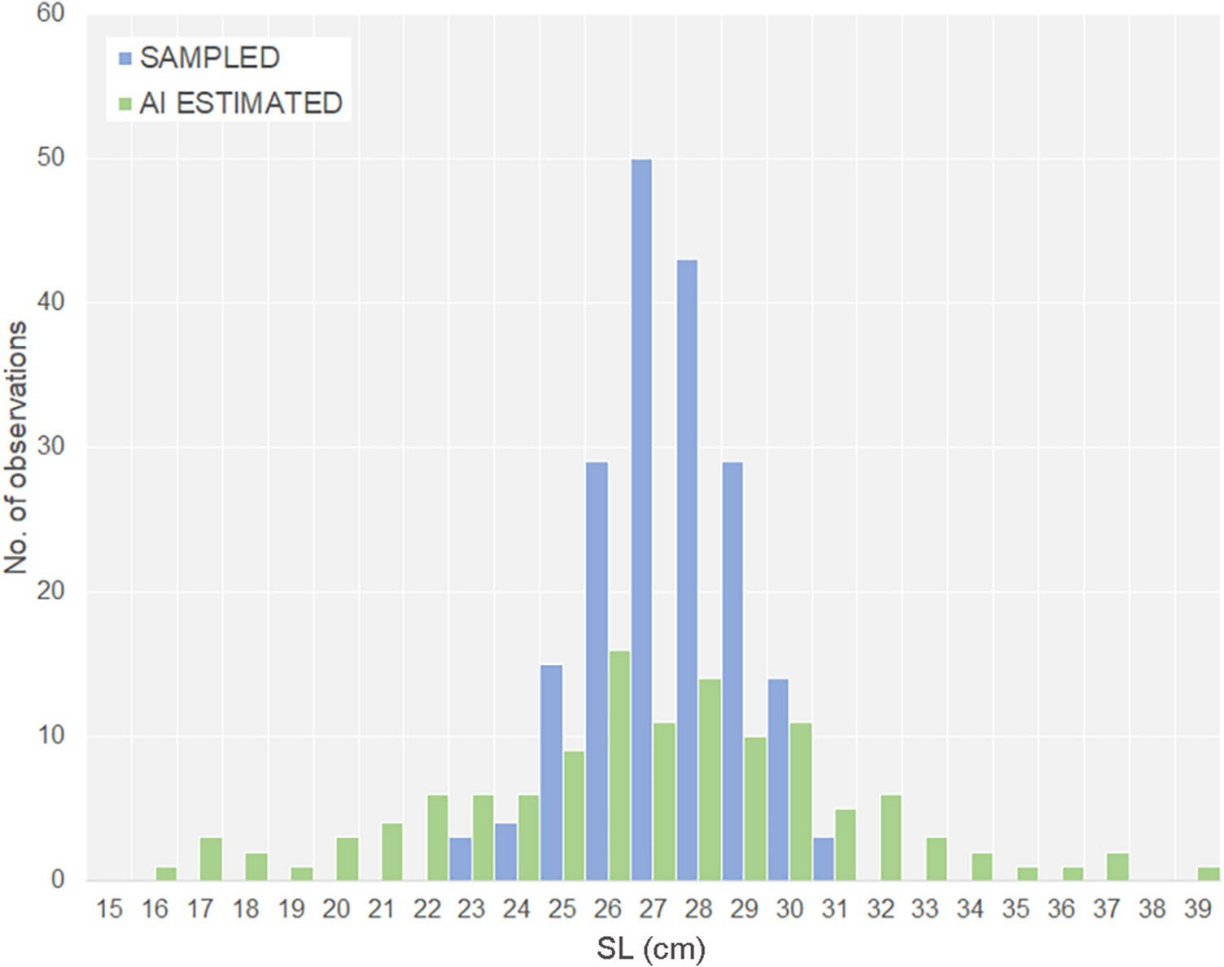
Standard-length histograms for sampled versus AI estimated distributions (Source: R version 4.2.1).

Both distributions behaved as normal curves (Shapiro = 0.9, p = 0.07; Shapiro = 0.99, p = 0.59, respectively for Sampled and AI estimated), but variances were not homogeneous (Levene test p = 0.00), as evident from the histograms in Fig. 2 However, the most representative classes (26, 27 and 28 cm bins) were found to be the same from both actual and AI measurements.

The AI estimated distribution showed a greater dispersion (i.e., more stretched) compared to the sampled one, which resulted more clustered. On the other hand, the means were not significantly different (F Welch test = 3.90, p = 0.05).

**Figure 3:**
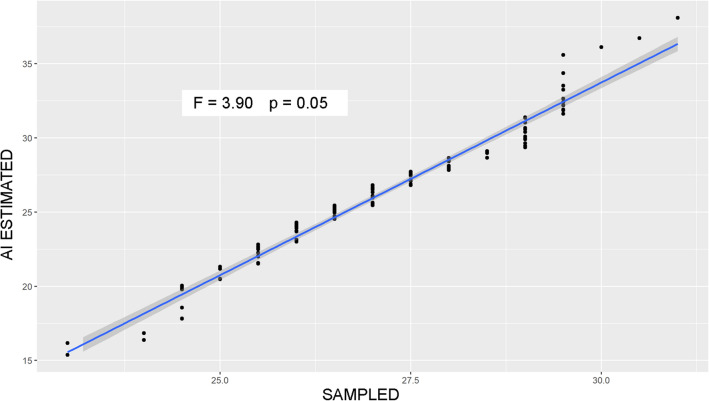
Q-q plot of sampled versus AI estimated fish standard length distributions (Source: R version 4.2.1).

The q-q plot shown in Fig. 3 (where the x-axis represented the estimated quantiles for Sampled dataset and the y-axis those for AI estimated) showed that the two dataset batches did not come from populations with a common distribution, and that differences were higher for lengths over 30 cm and below 25 cm (significantly higher and lower in AI estimated, respectively). Thus, the two distributions showed different tail behavior, while the central tendency overlapped.

## Discussion

With the effects of climate change looming on the horizon and the coming demand for fish that these changes will bring, aquaculture will be key. However, the need to accurately estimate fish biometrics is paramount for achieving appropriate feeding, promoting healthy growth, and improving breeding efficiency in fish farms^20^. Precision aquaculture and constant fish monitoring through camera-based image acquisition together with AI perfectly meet the needs of modern sustainable aquaculture. Precision aquaculture helps to reduce the environmental footprint and improve competitiveness and sustainability by increasing fish productivity and reducing operating costs and overfeeding, potentially allowing aquaculture to be a viable resource at present and in the future.

In recent years, computer vision has gained importance as a non-invasive method for the estimation of fish size classes in the precision farming approach. Computer vision-based monitoring uses stereoscopic cameras to obtain valuable images directly from the containment farming methods^21^. Nevertheless, one of the limitations to the widespread use of precision aquaculture technology is the employment of a large number of sensors, like integrated multiparametric probes used for water analysis, which makes the initial operational costs quite substantial. On the other hand, the cost of the computer vision systems (e.g., stereo cameras and hardware) is moderate, and the practicability is validated, providing a feasible methodology for assessing fish status in aquaculture^7^. The use of computer vision systems together with integrated multi-parametric probes is particularly suitable in offshore finfish mariculture, where good water quality and visibility allow for optimal performance. Despite this, there are still a few issues to deal with when using the computer vision approach: first, the light conditions and distortions of the underwater environment can hinder discerning fish from the background; second, fish are free to move in the water column, resulting in occlusion issues, making it arduous to detect and measure^7^.

The image acquisition device tested in this study proved to be practical and applicable to commercial mariculture production. The tool showed that the fish image features selected by the AI criterion were correctly identified; the proposed fish weight and length estimation method worked well as the average difference between automatic and sampling measurements only deviated by about ± 3%, and standard length distributions were found to be similar. Although as a prototype module, the monitoring buoy was tested on a commercial scale and could provide farmers with an accurate and automatic tool for the estimation of size and weight, capturing high-quality digital stereo images of fish. The images are automatically analyzed by an AI algorithm, which determines the size distribution classes of the fish. At the same time, the system records water quality parameters (temperature, dissolved oxygen, pH) through the multi-parametric probe, storing them on a cloud database. In this scenario, where AI replaces human labor, time spent manually measuring fish would be potentially usable time for other activities in the farm, thus improving productivity. The whole system is composed of hardware (the buoy, on which the stereo camera and probe are mounted), the mobile data transmission system (which directly shares the images on the cloud), and software (AI and geometric algorithm for automated detection and measurement of fish). The cost of the device is around 3500 euros for the basic version, making it an affordable tool, compared to other engineering solutions currently available on the market. Furthermore, the available commercial tools for fish length estimations require a considerable part of the analysis to be performed manually by human resources, unlike the proposed device, for which once the system is fine-tuned, no human control is needed. Recently, some start-ups have been trying to create automated tools based on AI algorithms to estimate the fish length and weight. However, to the authors’ knowledge, all these attempts are in a preliminary stage, and/or most of them are tested under experimental laboratory conditions. Other companies have developed similar devices and offer advisory services to farmers, although they do not sell the equipment to be used by farmers themselves; this could represent a limitation on the freedom in the management of fish farms.

Most of the Mediterranean aquaculture companies are considered small-scale enterprises when compared to the large salmon farms of northern Europe. Nonetheless, these small-scale companies have the constant need to increase their production capabilities in a sustainable and economically valuable way due to high demand. As such, new solutions should be found for a better and more sustainable estimation of fish length and weight for small and micro-enterprises that are not able to invest large funds for innovative technologies. This study demonstrated that machine vision and neural network model could be used for accurate body length predictions in cultured gilthead seabreams—one of the two most important species farmed in the Mediterranean Sea—and potentially in other marine and freshwater species. Moreover, the system could be used as a reliable, relatively cheap, stress-free, and accurate instrument for monitoring and estimating fish biomass during on-growing phase. Our tool has achieved excellent results in terms of accuracy (estimation accuracy reached 97%), efficiency, and automation, and despite being a prototype module, it is quite affordable. Continuous measuring of water parameters, integrated with machine vision assessments, can help predict precise feed volumes for fish growth at different stages. Additionally, creating a database will allow historical data to be run through forecasting models to anticipate how parameters affect fish production.

Further steps for the optimization of the device will include the improvement of the buoy system and the quality of the stereoscopic photos. Such upgrades will allow for better results in assessing the size distribution and biomass of stocks, making it appealing to other farming methods, such as land-based aquaculture (raceways, ponds, and tanks), and different fish species. At present, most efforts in using computer vision systems in aquaculture are associated with the shaping, behavior, counting, or body size estimation of fish (mostly length)^11–14^, with few studies attempting to show that biomass can be estimated more accurately when measurements of fish in dimensions other than length, are available, such as girth or view area^4,15–17,22^. We are currently working to find a suitable way to estimate fish weight by integrating data information from the fish side view area into the model. As further steps, integrated computer vision and water quality information could be used by AI to deliver early warnings, such as heat waves or oxygen fluctuations, while reducing production losses. Building such data sets will help mitigate low yields and therefore waste, as a way to improve overall sustainability. Finally, an intuitive user interface will be developed for easy use by non-AI and computer vision experts.

## Methods

### Farming site characteristics

The study was carried out at the “Maricoltura e Ricerca Società Cooperativa” fish farm (43°03′34.0"N 9°50′19.4"E) approximately 0.17 nautical miles off Capraia Island (Tuscany Region, Northern Tyrrhenian Sea, Italy). The farming site area is characterized by a rocky bottom, water depth of about 35–40 m, dissolved oxygen concentration of 5.95 ± 0.30 mg/L (mean ± SD), and annual surface water temperature of 24.13 ± 0.53 °C (mean ± SD). The facility consists of ten circular sea cages, eight 2400 m^3^ cages dedicated exclusively to gilthead seabream and European sea bass (*Dicentrarchus labrax* L.) farming, and two 900 m^3^ cages also devoted to experimental trials.

### Smart buoy and stereoscopic camera characteristics

**Figure 4:**
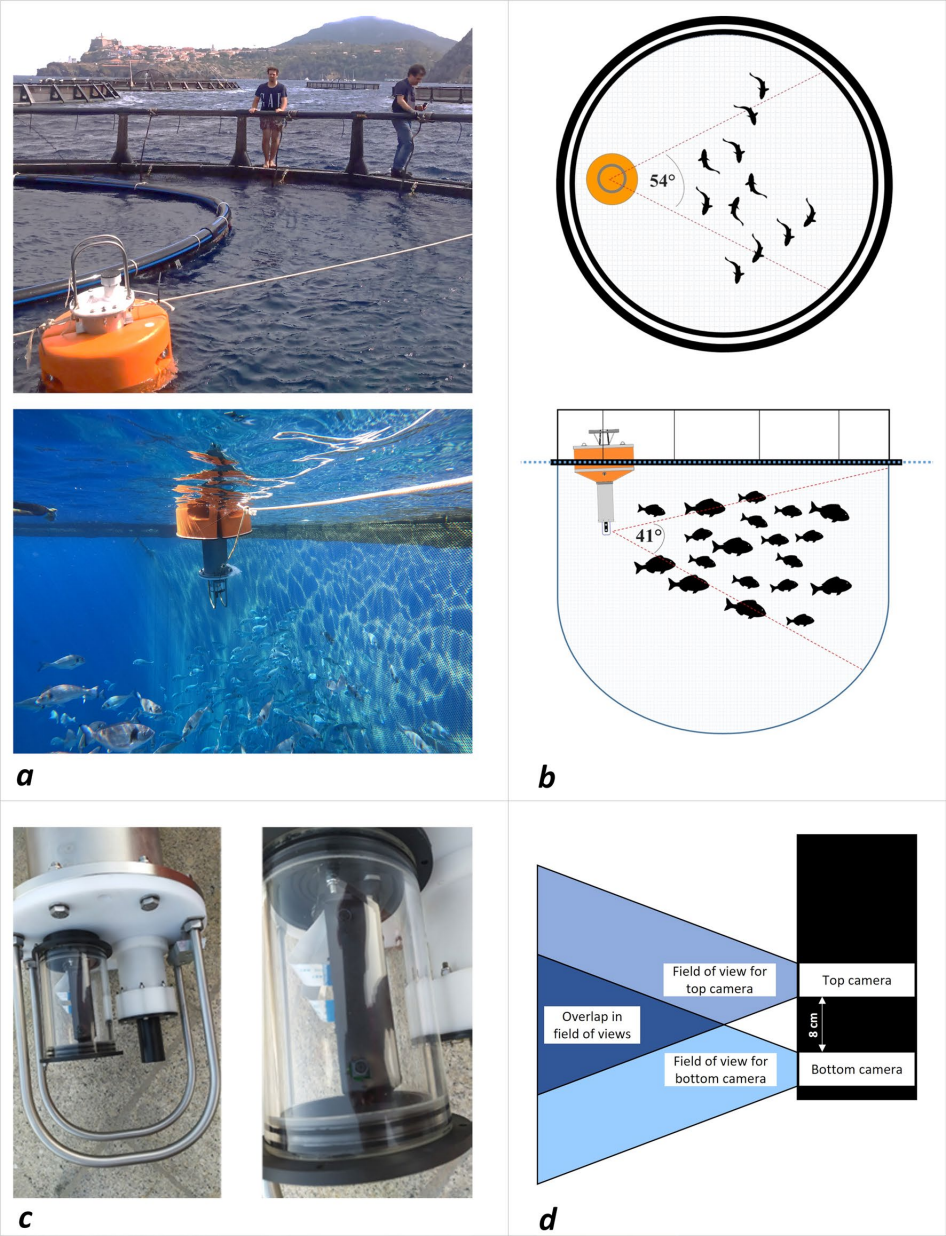
(**a** ,**b**) Photos and images of buoy positioning inside the sea cage (Photos by N. Tonachella and M. Martinoli). (**c**) Close-ups of the stereo camera and plexiglass waterproof housing (Photo by F. Capoccioni), and (**d**) diagrams of the stereo camera's fields of view.

The smart buoy was composed of a 1.2 m × 0.2 m stainless steel cylinder, fixed to a 0.6 m wide float (Fig. 4a). The device was equipped with a lithium battery pack, a 4G network connection router, a multiparametric probe (measuring temperature, pH, and dissolved oxygen), and a stereo camera (Fig. 4c). The buoy was anchored inside a commercial scale farming cage for underwater image recording and tied by ropes to the floating collar of the cage. The integrated stereo camera was placed at a depth of about 0.7 m and sealed in a waterproof housing (plexiglass cylinder). The mounted camera was an 8MP Arducam synchronized stereo camera consisting of two 8MP IMX219 camera modules capable of taking pictures simultaneously thanks to a connection on Raspberry Pi (Table 2). The two-camera lenses were spaced 8 cm apart on the vertical axis and the device was oriented towards the cage net (Fig. 4a–d). The smart buoy transmitted over a mobile network to a cloud-based site where images and data were stored. The images were accessible for download on a personal computer. During the trial period (3 months, from 1st July to 30th September 2021), the fish cage hosted about 4000 gilthead seabreams (mean weight 606 ± 103 g). Daily feeding and all routine farm procedures were performed by the farmers’ operators during the entire trial period. At the end of the experiment, 200 fish were collected and standard length (SL) and weight (W) were recorded to determine the length–weight relationship curves and compare the results obtained from the image analysis to the actual size of the fish.

**Table 2:** Technical characteristics of the stereo camera.

Image sensor		Lens assembly	
Sensor model	IMX219	Interchangeability	No
Shutter type	Rolling shutter	f/no	2.8
Active pixels	3280 (H) × 2464 (V)	Focus type	Fixed focus
Resolution	8MP × 2	Field of view	54°H × 41°V

### Image data calibration and analysis

There are a variety of approaches for geometric camera calibration^23^. Many of them have in common the use of markers or patterns, which represent visible and distinguishable object points. These object points and their corresponding image points are then used as observations to determine the parameters of the camera model(s) and the relative orientation(s)^24^. In the underwater environment, the calibration must model and compensate for the refractive effects of lenses, the housing port, and the water medium^10^. Computer vision approaches often use 2D test fields in the form of chessboard targets. Usually, the 2D calibration technique employs a planar calibration pattern of alternating black and white squares to determine the intrinsic and extrinsic parameters of the camera^25^.

#### Camera calibration and model fitting

**Figure 5:**
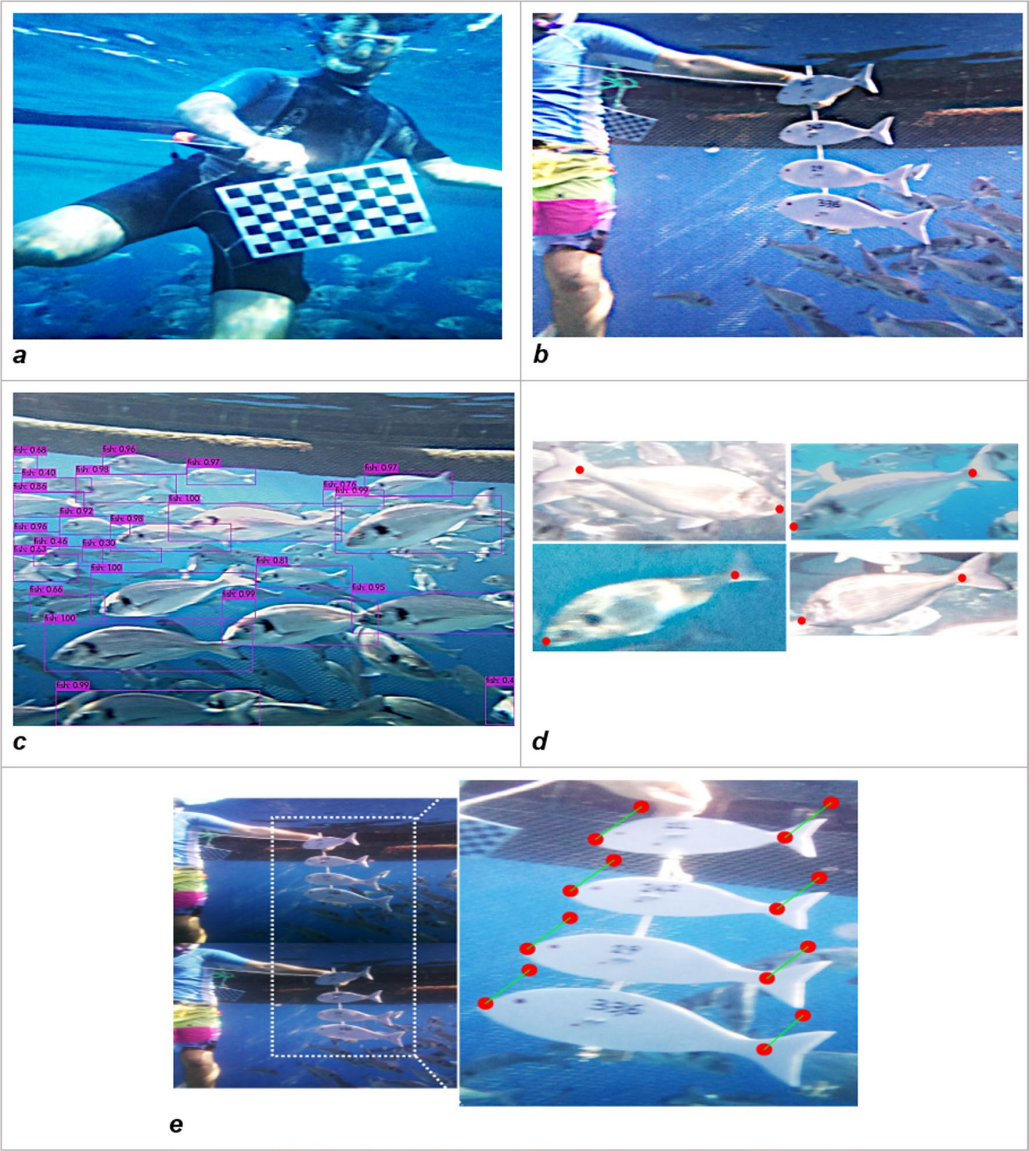
(**a**) Underwater image of the chessboard pattern used for calibration of real pixel size. (**b**) Example of fish silhouettes validation and error estimation in the cage. (**c**) Bounding boxes of automatic annotated fish with confidence thresholds. (**d**) cropped images of single fish and landmarks apposition. (**e**) translation example of fish silhouettes comparing the two stereo-camera images (red dots represent the same landmark from different lenses).

In this study, several replicate calibrations were performed in the water and in various orientations using a chessboard (270 × 190 mm) as a planar calibration pattern (Fig. 5). The corners of 15 squares were manually marked, each square measuring 27 × 27 mm. In this phase, the chessboard images were used to estimate the camera’s radial distortion parameters (Eq. 1, distortion matrix^26^), and to correct the refraction caused by the propagation of light through different substances^27^. Estimating the radial distortion coefficient helped to remove the barrel and cushion effects brought in by the camera and the housing.

1 $$\begin{gathered} \hfill \\ \begin{array}{*{20}c} {\left[ {\begin{array}{*{20}c} {f_{x} } & 0 & 0 \\ s & {f_{y} } & 0 \\ {c_{x} } & {c_{y} } & 1 \\ \end{array} } \right]} & {\begin{array}{*{20}l} {\frac{{\left[ {c_{x} ~c_{y} } \right] - {\text{Optical~center~}}\left( {{\text{the~principal~point}}} \right),{\text{~in~pixels}}~}}{{~\left( {f_{{x~}} ~f_{y} } \right) - ~{\text{Focal~length~in~pixels}}}}} \hfill \\ {f_{x} = {\raise0.7ex\hbox{$F$} \!\mathord{\left/ {\vphantom {F {p_{x} }}}\right.\kern-\nulldelimiterspace} \!\lower0.7ex\hbox{${p_{x} }$}}} \hfill \\ {f_{y} = {\raise0.7ex\hbox{$F$} \!\mathord{\left/ {\vphantom {F {p_{y} }}}\right.\kern-\nulldelimiterspace} \!\lower0.7ex\hbox{${p_{y} }$}}} \hfill \\ {F - {\text{Focal~length~in~world~units}},{\text{~expressed~in~millimeters}}} \hfill \\ {\frac{{\left( {p_{{x~}} ~p_{y} } \right) - ~{\text{Size~of~the~pixels~in~world~units}}}}{{{\text{s}} - {\text{Skew coefficient}},{\text{ which is non}} - {\text{zero if the image axes are not perpendicular}}}}} \hfill \\ {{\text{s}} = f_{x} \tan \alpha } \hfill \\ \end{array} } \\ \end{array} \hfill \\ \end{gathered}$$

In a second phase, the stereo images (one pair of images per photo shoot) were properly marked with 4 pairs of reference points (landmarks) in different positions of the board (corners) to estimate the translation of each pixel of the board between the two stereo images; the relative translation of the target in the two stereo images is directly related to the distance between the camera and the target itself (Fig. 1). The closer the target is to the camera, the greater the translation of the target between the two stereo images. This was key information to correctly estimate the actual target size in pixels. A total of 52 images and 208 single measurements from the calibration chessboard were used to compute the relationship.

#### Measurement error estimation

In order to estimate the measurement error (mean absolute percentage error – MAPE), 17 photos of plastic fish silhouettes of four known different sizes were taken and processed (standard length of 22.0–24.2–29.0–33.6 cm each, Fig. 5). The images were captured by placing the targets in front of the camera, at increasing distances. The known lengths of the fish silhouettes were compared with the estimated lengths of the AI to calculate the error in cm and as a percentage of the fish body length.

#### AI automatic fish recognition, landmarks positioning, and fish length measurements

**Figure 6:**
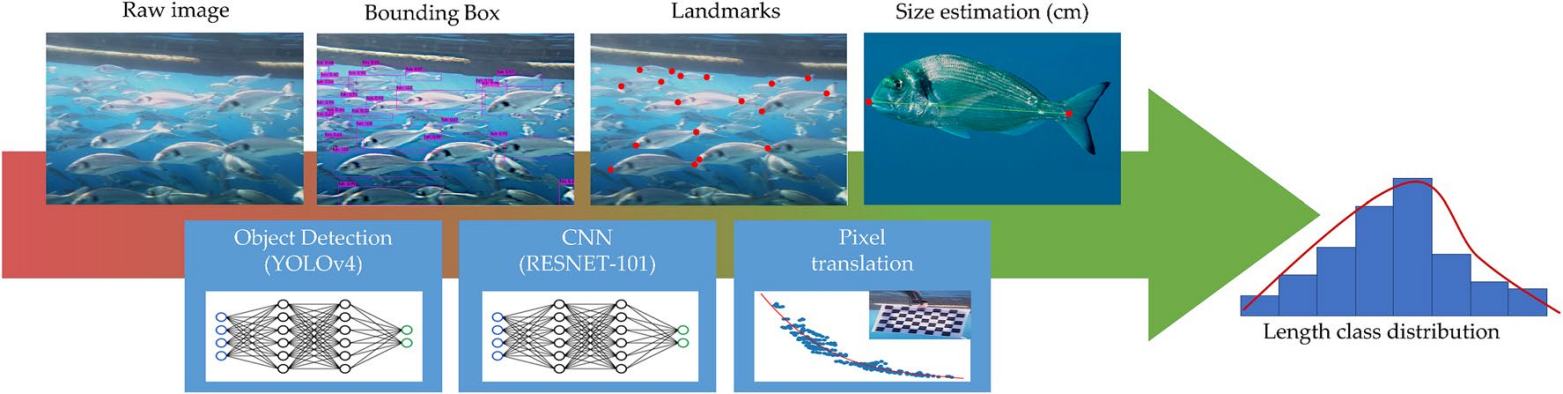
The overall process of the proposed method: automatic AI fish recognition and size estimation.

During the image acquisition stage, the fish swam freely within the cage, without being oriented along any of the x–y axes of the camera plane. For fish body length estimation, a complex AI pipeline was designed (Fig. 6). The pipeline was split into smaller packages to break down the final pipeline task into its components and thus simplify and manage the analysis more efficiently.

The raw stereo images were fed to an improved Convolutional Neural Network (CNN) called You Only Look Once (YOLO) v4^28^. As an excellent one-stage detection algorithm, the YOLO series algorithm has high detection accuracy, fast detection speed, and is widely used in various target detection tasks. Several studies applied this algorithm for detection purposes: Tian et al.^29^ used improved YOLOv3 to detect apples at different growth stages; Shi et al.^30^ proposed a YOLO network pruning method, which can be used as a lightweight mango detection model for mobile devices; and Cai et al.^31^ proposed an improved YOLOv3 based on MobileNetv1 as a backbone to detect fish. In this study, YOLOv4 CNN was trained with 1400 properly annotated images (Training: n = 1120, validation: n = 280), collected from the Open Image dataset (Open Images Dataset V6 – storage.googleapis.com) and from the field, to locate individual fish within the image using bounding boxes. The training was carried out for 6000 iterations and reached a CIoU Loss = 1.5^32^ and an mAP = 87%^33^. In a second step, each bounding box was used to obtain the individual image of the fish, which was then entered into a well-known CNN, RESNET-101 (RES101)^34^, optimized for image recognition. The training of RES101 was carried out in Pytorch^35^ using the transfer learning technique as proposed by Monkman et al.^36^. Moreover, as for the individual fish location, the automatic landmarks detection was achieved using CNN, RESNET-101, with the last layer being modified to detect two landmarks (the snout tip and the base of the middle caudal rays) on the fish shape. The training (n = 8960) and test dataset (n = 3840) were obtained from 200 field pictures where each relevant individual fish were extracted and manually annotated with the landmarks required. Each image was then fed into an augmentation algorithm that generated 64 augmented images of different levels of scale, noise, rotation, translation, and brightness. The process generated the final image dataset of 12,800 pictures. The training was carried out for 100 epochs and generated an MSE = 0.23 (Mean Square Error between the predicted and true landmarks. This automatic landmark positioning allowed the algorithm to measure the fish length in pixels by counting the pixels between the two points. Finally, the length unit was transformed from pixels to centimeters, using the translation information derived from the chessboard target images during the calibration phase: the further a target is placed from the cameras, the less it translates between a pair of stereo images and vice versa. The extent of this translation can be measured both as an angle (the parallax angle) or as a distance in pixels between the same point in the two stereo images (Fig. 5e); the same technique is used in the parallax method for estimating the distance of the stars^37^. Since the size of the chessboard was known, the translation in pixels of the chessboard’s landmarks was then plotted against their corresponding ratio between length in cm and length in pixels; the fit model generated has become more accurate the more stereo images were tested in different positions within the camera's field of view.

The observation points obtained by the described computation were then entered into a polynomial Ridge Regression algorithm which produced a function minimizing the Residual Mean Square Error^38^. This model was finally used to estimate the micron/pixel converting factor of a given fish, through which the final length in cm is obtained. The distribution of standard length values achieved employing the AI algorithm (n = 124, called “AI estimated”) was compared to the one directly measured on a subsample of 190 fish (called “Sampled”) collected in the same cage where stereo images were taken. The fish employed in this study were harvested by the farm staff and destined for sale in large retailers, as they pertained to the fish farm. A random sub-sample of all catches from the cage was given to the researchers for comparative analysis. Therefore, we treated fish already sacrificed, in line with the current national legislation for farmed animals. The length comparison was carried out using a quantile–quantile (q-q) plot, i.e., a graphical technique for determining if two data sets come from populations with a common distribution (Fig. 3). If this assumption is true, the points in the scatterplot should fall approximately along the 45° reference line plotted in the graph. The greater the deviation from this baseline, the greater the evidence that the two data sets come from populations with different distributions. The Shapiro–Wilk test (alpha level = 0.05) was performed to test for the data set's normality, whereas the Leven test (alpha level = 0.05) was carried out to assess the homogeneity of variance between the two distributions. Then, the Welch-F test (alpha level = 0.05) was used to compare the distribution means in the case the homogeneity of variances was violated.

Finally, a length-to-weight relationship (LWR)^39^ (Eq. 2) was determined using body weight (g) and standard length (cm) measurements from sampled fish (n = 198):

2 $$W = aL^{b}$$

where W is the body weight of the fish, a is the intercept linked to body shape, L is the standard length, and b is the exponent interrelated to variations in body shape. The obtained LWR relationship was used to calculate the fish weight from length values derived from the images processed by the AI^40^(fish were collected on the same day the photos were taken by the stereo camera).

The experimental activities involving animals conducted in this study, including their ethical aspects, were approved by the Animal Welfare Body of the CREA Centre for Animal production and aquaculture (authorization n. 86670 of 23/09/2021). No human experiments were performed, nor were human tissue samples used. All the people depicted in the images represent the authors of the study during the logistical organization of the calibration tests. Informed consent was obtained from all individual participants both for participation in the study and for the publication of identifying information/images in an online open-access publication.

## Data Availability

The data that support the findings of this study are available from the corresponding author upon reasonable request.
